# MiR-21-5p Induces Pyroptosis in Colorectal Cancer *via* TGFBI

**DOI:** 10.3389/fonc.2020.610545

**Published:** 2021-02-05

**Authors:** Rilei Jiang, Xiaolei Chen, Shaohua Ge, Qin Wang, Yichang Liu, Haijun Chen, Jiatuo Xu, Jiang Wu

**Affiliations:** ^1^ School of Basic Medicine Science, Shanghai University of Traditional Chinese Medicine, Shanghai, China; ^2^ Department of Oncology, Kunshan Hospital of Traditional Chinese Medicine Affiliated to Nanjing University of Chinese Medicine, Kunshan, China; ^3^ First Clinical Medical College, Nanjing Medical University, Nanjing, China

**Keywords:** colorectal cancer, pyroptosis, programmed cell death, TGFBI, miR-21

## Abstract

Pyroptosis is a distinct form of programmed cell death in eukaryotic cells that has garnered increasing attention in cancer-related research. Moreover, although miR-21 has been reported as abnormally expressed in colorectal cancer, due to a lack of in-depth research on the transcriptional regulation mechanisms of miR-21, its clinical usage remains limited. Our study is the first, to our knowledge, to compare the clinical manifestations and laboratory phenotypes associated with miR-21-3p and miR-21-5p. Morphologically, the transfection of miR-21-3p or miR-21-5p inhibitors, as well as miR-21-5p mimics into HCT-116 and HT-29 cell lines, induced cell death. Surprisingly, overexpression of miR-21-5p induced cell death more strongly than its knockdown. Mechanistic studies of miR-21-5p overexpression revealed that various inflammatory factors including IL-1β and IL-18 were released, while pyroptosis-associated mRNAs were upregulated and proteins were activated. Moreover, miR-21-5p was found to act as a downstream factor to significantly and directly regulate transforming growth factor beta-induced (*TGFB1)*. Specifically, miR-21-5p overexpression caused downregulation of *TGFBI*, which may have led to pyroptosis. Collectively, we revealed that miR-21-5p induces pyroptosis in colorectal cancer *via TGFBI* regulation, thereby providing important mechanistic insights into its antitumor effects and expanding its potential for clinical applications.

## Introduction

Colorectal cancer (CRC) was among the most common cancers diagnosed in men and women in 2019 (1); while the difficulties associated with its early detection, as well as its strong tendency for metastasis, and resistance to conventional therapies (2–4) makes its treatment challenging. In fact, in 2015, approximately 376,300 new CRC cases were reported with an estimated 191,000 associated mortalities in China alone (5).

Pyroptosis is recognised as a distinct form of programmed cell death in eukaryotic cells (6), and has recently become a new frontier in cancer-associated research (7). Pyroptosis is characterised by cell swelling with large bubbles emerging from the plasma membrane (8). In cells undergoing pyroptosis, mature caspase-1 cleaves a protein from the gasdermin family and activates inflammatory cytokines, such as IL-18 and IL-1β7, which recruit inflammatory cells and expand the inflammatory response (9).

Programmed cell death (PCD) depends on specific genes encoding proteins associated with various signalling pathways or functions (8). Regarding pyroptosis specifically, studies on its underlying mechanisms have primarily focused on non-coding RNAs (ncRNAs), such as long non-coding RNAs (lncRNAs) and microRNAs (miRNAs), that are upstream of both the canonical and noncanonical inflammasome pathways. For instance, knockdown of lncRNA RP1−85F18.6, which represents a tumour promoter in CRC, decreases the expression of ΔNp63 and inhibits tumour growth and metastases by inducing pyroptosis (10). Moreover, metformin has been reported to induce pyroptosis of oesophageal carcinoma cells through the miR-497/PELP1 axis (11). MiRNA-214 also induces pyroptosis in glioma cells by targeting caspase 1 (12). Although some studies have reported on the role of pyroptosis in various cancers, compared to the detailed mechanisms reported for other types of PCD, such as apoptosis, those related to pyroptosis are lacking with only the proverbial tip of the iceberg exposed. Hence, the detailed mechanism underlying pyroptosis in cancer requires further elucidation.

Generally, miRNAs repress the expression of target genes by binding to complementary sequences at the 3ʹUTR of mRNAs to prevent their translation (13). Since the discovery of miRNAs two decades ago, their biological functions have been extensively studied (14), particularly in the field of cancer. miR-21, specifically, has been extensively studied in various diseases, including renal fibrosis (15), hepatocellular carcinoma (16), atherogenesis (17), and cancer. In fact, miR-21 has been reported as overexpressed in many cancerous tissues compared to normal tissues and was briefly recognised as an onco-predictive biomarker (18, 19). However, due to a lack of in-depth understanding of miR-21 transcriptional regulatory mechanisms, target genes, and associated mechanisms, the clinical application of miR-21 is limited.

As a precursor, pre-miR-21 is processed by an RNase III complex to generate two mature miRNAs, namely, miR-21-3p and miR-21-5p (20). Currently, reports on CRC have primarily focused on miR-21-5p (guide strand), with the most commonly reporting pathways being those associated with the miR-21-5p/PTEN-regulatory networks which seem to intersect with the PI3K/Akt pathway (21, 22). However, minimal research has been undertaken on miR-21-3p in CRC or on the comparison of miR-21-3p and miR-21-5p networks in the context of CRC.

In this study, we first compared the clinical manifestations and laboratory phenotypes of miR-21-3p and miR-21-5p in CRC cells. The overexpression of miR-21-5p was found to induce cell death more strongly than its knockdown. Meanwhile, mechanistically, inflammatory factors were observed to be released, thereby activating pyroptosis. Downstream analysis further determined that miR-21-5p directly downregulates *TGFBI* leading to subsequent pyroptosis.

## Materials and Methods

### Reagents, Cell Lines, and Clinical Tissues

HCT-116 and HT-29 cell lines were purchased from Shanghai Zhong Qiao Xin Zhou Biotechnology Co. Ltd. (Shanghai, China). The 293T cell line was provided by Stem Cell Bank, Chinese Academy of Sciences (Shanghai, China). Human colonic epithelial cells (HCECs) were kindly provided by the Traditional Chinese Medicine Hospital of Kunshan (Kuanshan, China). The HCECs, HCT-116 and 293T cells were cultured in DMEM-high glucose (HyClone, USA) supplemented with 10% foetal bovine serum (FBS; Gibco, South America) and 1% penicillin/streptomycin (P/S; Gibco, Australia). HT-29 cells were cultured in McCoy’s 5A (Gibco, Australia) with 10% FBS and 1% P/S.

Five colon cancer patients were random selected from the Department of Oncology, Kunshan Hospital of Traditional Chinese Medicine Affiliated with Nanjing University of Chinese Medicine. Patient demographic information is provided in **Supplementary Table 6**. From each patient, tissues were obtained from colonic tumours as well as from adjacent normal tissue (2 cm away from tumour). All protocols were approved by the Ethics Review Committee of Kunshan Hospital of Traditional Chinese Medicine (Approval No: KZY2017-16).

### Plasmid Construction and Cell Transfection

The mimics and inhibitors for hsa-miR-21-3p, hsa-miR-21-5p, and non-specific control (NC; **Supplementary Table 1**) were constructed by Genomeditech Co. (Shanghai, China). Three individual short hairpin RNA plasmids for *TGFB1* (sh-TGFBI-1, sh-TGFBI-2, and sh-TGFBI-3) and a negative control (sh-NC) (**Supplementary Table 2**) were purchased from Genomeditech Co. (Shanghai, China). The wild-type and mutant type dual-luciferase reporter plasmids (**Supplementary Table 3**), constructed using pmirGLO vector, were also purchased from Genomeditech Co. (Shanghai, China). The plasmids were transfected according to the manufacturer’s instructions using Lipofectamine 2000 (Invitrogen, USA).

### qRT-PCR

Total RNA was extracted using TRIzol reagent (Invitrogen). Next, miRNA quantification was performed using miRNA First Strand cDNA Synthesis (Stem-loop Method; Sangon Biotech, Shanghai, China) and the microRNA qPCR Kit (SYBR Green Method; Sangon Biotech, Shanghai, China). Quantification of mRNA was performed as previously described (23). The amount of cDNA template used in each reaction was between 20~200 ng (10 μl). The reaction conditions were as follows: denaturation at 95°C for 5 min (Hot-Start) followed by 40 cycles of amplification at 95°C for 10 s and 60°C for 30 s. Ct values between 20 and 30 were regarded as valid results. The primer sequences used are listed in **Supplementary Table 4**. The value of 2^-ΔΔCT^ was used for analysis. *GAPDH* was used as internal control mRNA, U6 was used as an internal control for miRNA.

### Western Blot Analysis

The proteins were isolated using a radio-immunoprecipitation assay (RIPA) protein extraction reagent (Beyotime, Shanghai, China) with 1% phenylmethanesulfonyl fluoride (PMSF, Beyotime). Next, proteins were suspended in 10%–15% SDS-PAGE gels (Beyotime) and transferred to PVDF membranes (Invitrogen), followed by incubation with the following primary antibodies: Caspase-1 (1:1,000, #3866, CST, USA), cleaved Caspase-1 (1:1,000, #4199, CST, USA), GSDMD (1:1,000, #93709, CST, USA), cleaved GSDMD (1:1,000, #36425, CST, USA), interleukin (IL)-1β (1:1,000, #12703, CST, USA), cleaved IL-1β (1:1,000, #83186, CST, USA), TGFBI (1:500, 10188-1-AP, Proteintech, Wuhan, China), and β-actin (1:2,000, #4970, CST, USA).

### Cell Counting Kit-8 Assay

The viability of CRC cells was assayed using the Cell Counting Kit-8 (CCK-8; Dojindo, Beijing, China) assay. After transfecting cells with mimics or inhibitors, 5 × 10^3^ cells/well were seeded in a 96-well plate, with 100 μl of suspension in each well. After culturing for 24 h, the cells were stained with CCK-8 solution and incubated for 30 min. Cell viability was calculated after measuring the absorbance at 450 nm.

### TUNEL Assay Analysis

Cells cultured in confocal dishes were fixed with 4% paraformaldehyde for 20 min and were subsequently stained with the TUNEL kit on ice followed the manufacturer’s protocol (One-step TUNEL Detection Kit, Beyotime, Shanghai, China). TUNEL-positive cells were counted under a laser scanning confocal microscope (Leica, TCS SP5, Germany).

### Flow Cytometric Analysis

The FITC-Annexin V apoptosis detection kit (MEILUN, Dalian, China) was used to detect the cell apoptosis ratio according to the manufacturer’s protocol. In brief, the CRC cells were double-stained with propidium iodide (PI) and Annexin V for 30 min in the dark. The cell apoptosis ratio was then detected using a FACS ARIA II SORP Flow Cytometer (BD, USA).

### ELISA Assays

IL-1β and IL-18 levels in the cell culture medium were detected by using a Human IL-1β High Sensitivity ELISA kit (EK101BHS-01, Multi Science, China) and Human IL-18 ELISA kit (EK118-01, Multi Science, China), according to the manufacturer’s instructions. The optical density was measured at a wavelength of 450 nm, while measurement at 570 nm were screened as an internal control.

### Immunofluorescence

Cells were treated with 4% buffered paraformaldehyde for 20 min at room temperature. Next, 1% BSA and 0.1% Triton-X were used to block the membrane for 2 h at room temperature. The membranes were then treated with primary antibodies against ASC/TMS1 (1:200, 10500-1-AP, Proteintech, Wuhan, China) or TGFBI (1:200, 10188-1-AP, Proteintech, Wuhan, China) at 4°C overnight. The secondary antibody (anti-rabbit IgG (H+L), F(ab’)2 fragment (Alexa Fluor^®^ 488 Conjugate, #4412, CST, US), or anti-rabbit IgG (H+L), F(ab’)2 Fragment (Alexa Fluor^®^ 594 Conjugate, #8889, CST, US) were added to the cells for 1 h at room temperature. Cells were incubated with Antifade Mounting Medium with 4’,6-Diamidine-2’-phenylindole dihydrochloride (DAPI; Beyotime, Shanghai, China) to detect ASC specks or TGFBI expression and distribution using a laser scanning confocal microscope (Leica, TCS SP5, Germany).

### Dual-Luciferase Reporter Assays

The fragments of wild-type and mutant type *TGFBI* were amplified by PCR and cloned into a pmirGLO Dual-Luciferase miRNA Target Expression Vector (Promega, USA) to generate pmirGLO-TGFBI-wt and pmirGLO-TGFBI-mut reporter vectors. The above vectors with miR-21-5p mimics and NC mimics were co-transfected into 293T, HCT-116, and HT-29 cells by Lipofectamine 2000 (Invitrogen, US). Finally, the cells were lysed using the Dual-Luciferase Reporter Assay System (Promega, USA) and luciferase activity was detected. Renilla luciferase activity was used as an internal control.

### Statistical Analysis

Experimental groups were compared with controls using the Student unpaired, two-tailed *t*-test. Multiple groups were expressed as mean ± SD and analysed by Kruskal-Wallis analysis. P < 0.05 was considered as statistically significant. Graph Pad Prism 6.0 was used for statistical analysis.

## Results

### miR-21-3p and miR-21-5p Expression in CRC and Model Establishment

**Figure 1:**
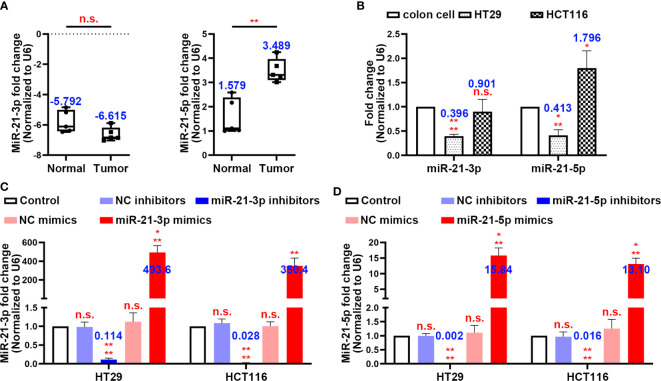
MiR-21-3p and miR-21-5p expression in colorectal cancer (CRC) and model establishment. **(A)** The expression levels of miR-21-3p and miR-21-5p in CRC patients’ tumours (n = 5) and normal tissues (n = 5). **(B)** The expression levels of miR-21-3p and miR-21-5p in normal colon cell, HT29, and HCT116. **(C, D)** Validation of miR-21-3p mimics/inhibitors **(C)** and miR-21-5p mimics/inhibitors **(D)** efficiency in HT29 and HCT116 cells as determined by qRT-PCR. The bars and error bars indicate the mean ± SD. n.s.p > 0.05, *p < 0.05, **p < 0.01, ***p < 0.005, ****p < 0.001.

By using clinical colon tissues from CRC patients’ tumours (n = 5) and adjacent normal tissues (n = 5), we found that the expression of miR-21-3p did not differ significantly between the normal and tumour tissues, while miR-21-5p was more highly expressed in the tumours (**Figure 1A**). Similarly, the expression of miR-21-3p was low in the CRC cell lines while that of miR-21-5p showed a high expression trend in the HT29 and HCT116 cells by PCR (**Figure 1B**). To further investigate the roles of these miRNAs in CRC, we constructed miR-21-3p/miR-21-5p mimics and inhibitor vectors. After transfection of these vectors, the expression levels of the two miRNAs changed as expected, mimicking the results described above (**Figures 1C, D**).

### Overexpression of miR-21-5p Induced Cell Death and Repressed Cell Viability in the CRC Cells Lines

**Figure 2:**
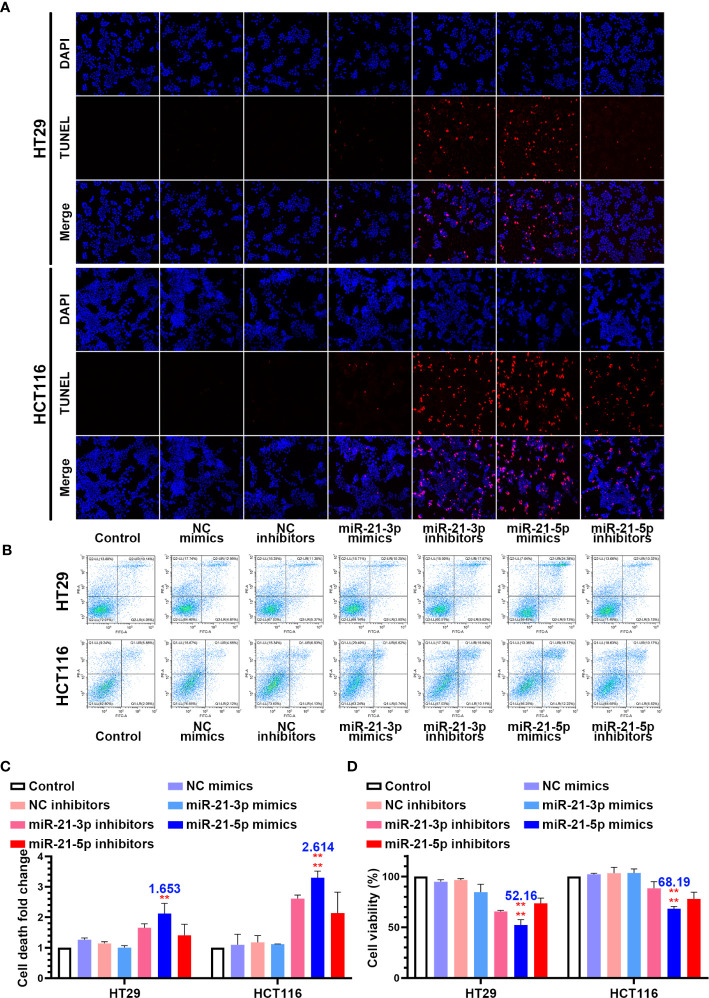
Overexpression of miR-21-5p induced cell death and repress cell viability in colorectal cancer (CRC) cells lines. **(A)** HT29 and HCT116 cells were transferred by miR-21-3p/miR-21-5p mimics and inhibitors, and cell death was determined by TUNEL stain assay. **(B, C)** Annexin V and PI staining flow cytometric analysis of cell death in miR-21-3p/miR-21-5p mimic and inhibitor transfected HT29 and HCT116 cells. **(D)** CCK-8 assay of HT29 and HCT116 cell viability changes with miR-21-3p/miR-21-5p mimics and inhibitors transfection. The bars and error bars indicate the mean ± SD. **p < 0.01, ****p < 0.001.

In an attempt to identify the precise roles of miR-21-3p and miR-21-5p in CRC cells, we transfected the miR-21-3p/miR-21-5p mimics and inhibitor vectors into HCT-116 and HT-29 CRC cells. TUNEL staining showed that transfection of miR-21-3p inhibitors and miR-21-5p mimics induced nuclear fragmentation in both the HCT-116 and HT-29 cells, while other transfections did not have an effect (**Figure 2A**). Flow cytometric analysis also indicated that the death rate of CRC cells was upregulated in the treated with the miR-21-5p mimic (**Figures 2B, C**). Meanwhile, the cell counting kit-8 assay showed that the miR-21-5p mimic reduced the cell viability while the other groups had less of an impact on the CRC cells (**Figure 2D**).

### Evidence that the miR-21-5p Pathway Induces Pyroptosis in CRC Cells

**Figure 3:**
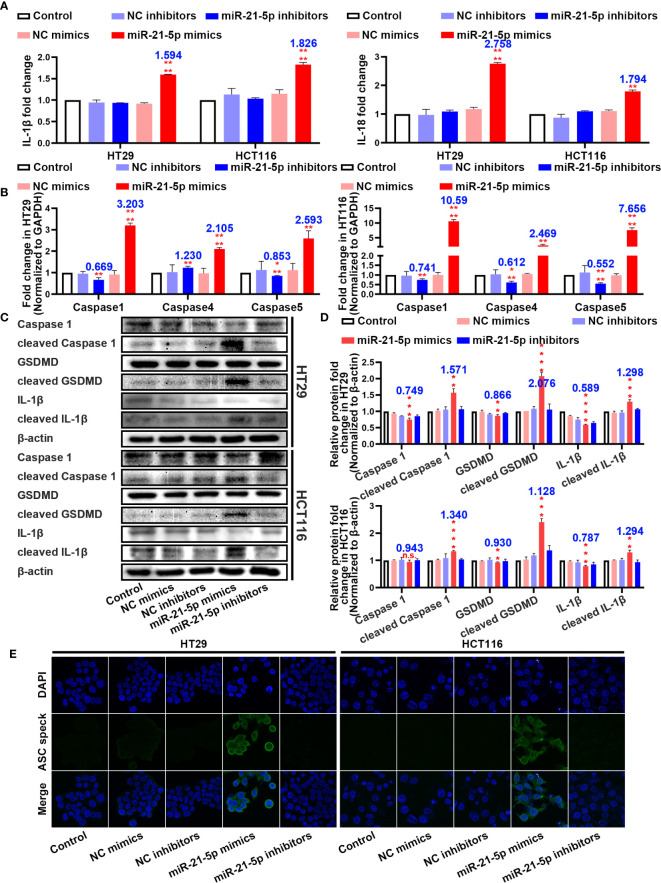
Pathway evidence of miR-21-5p induced pyroptosis of colorectal cancer (CRC) cells. **(A)** ELISA assay tested the IL-1β and IL-18 which released in HT29 and HCT116 cell culture mediums by the transfection of miR-21-5p mimics and inhibitors. **(B)** Pyroptosis-associated mRNA levels in HT29 and HCT116 cells after miR-21-5p mimics and inhibitors transfection as determined by qRT-PCR. **(C, D)** Expression of Pyroptosis-associated proteins in HT29 and HCT116 cells after miR-21-5p mimics and inhibitors transfection as determined by western blot analysis. **(E)** Immunofluorescence staining of ASC specks expression in miR-21-5p mimics and inhibitors transfected HT29 and HCT116 cells. The bars and error bars indicate the mean ± SD. n.s.p > 0.05, **p < 0.01, ***p < 0.005, ****p < 0.001.

In an attempt to determine the mechanism of cell death, we assayed inflammation-related indicators in the cell culture medium. Interestingly, the levels of IL-1β and IL-18 were significantly upregulated in the cells transformed with the miR-21-5p mimic compared to the other plasmids in both the HT29 and HCT116 cell lines (**Figure 3A**). As pyroptosis was the most immune-related cell death method, we detected pyroptosis-associated mRNAs and proteins. As suspected, the mRNAs related to pyroptosis were upregulated in the miR-21-5p transformed cells (**Figure 3B**) as were the cleavage products of caspase 1, IL-1β, and GSDMD (**Figures 3C, D**), indicating that the pyroptosis signalling pathway was activated. Furthermore, ASC oligomerization, as detect by immunofluorescence was apparent as ASC specks in cells transfected with miR-21-5p mimic (**Figure 3E**). It provided the final piece of evidence to pyroptosis.

### TGF-β1 Is a Potential Target of miR-21-5p

**Figure 4:**
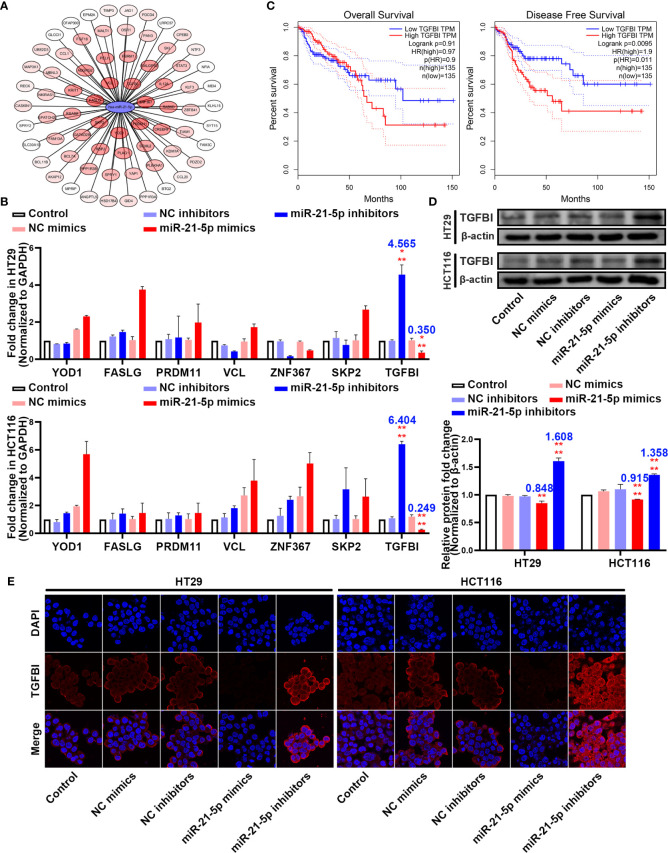
TGFBI is a potential target of miR-21-5p. **(A)** Bioinformatic prediction for candidate downstream targets of miR-21-5p. **(B)** Verification of miR-21-5p downstream targets by qRT-PCR in HT29 and HCT116. **(C)** Association of TGFBI expression with colorectal cancer (CRC) patient disease-free survival and overall survival. The areas between the same colour dotted lines represent 95% confidence interval for low/high TGFBI. **(D)** TGFBI expression in miR-21-5p mimics and inhibitors transfected HT29 and HCT116 cells as determined by western blot analysis. **(E)** Immunofluorescence staining of TGFBI expression in miR-21-5p mimics and inhibitors transfected HT29 and HCT116 cells. The bars and error bars indicate the mean ± SD. n.s.p > 0.05, **p < 0.01, ***p < 0.005, ****p < 0.001.

To further elucidate the molecular mechanism underlying how miR-21-5p exerts its effect on pyroptosis in CRC cells, we used bioinformatic tools to predict candidate downstream targets. After applying a target score ≥ 90, we identified 70 candidate targets (**Figure 4A**; **Supplementary Table 5**) and performed PCR to verify the target with the most potential using the more stringent inclusion criteria of a target score ≥ 98 in the HT29 and HCT116 cells (**Figure 4B**). The *TGFBI* mRNA was extremely changed following the variation tendency of complementary base pairing (**Figure 4B**). An analysis of data from the Cancer Genome Atlas (TCGA) demonstrated that higher *TGFBI* levels in CRC patients were always associated with poor overall survival and disease-free survival outcomes (**Figure 4C**). Moreover, western blotting analysis revealed that TGFBI expression increased as miR-21-5p decreased, and vice versa in the HT29 and HCT116 cells (**Figure 4D**). Meanwhile, immunofluorescence confirmed the western blot results (**Figure 4E**). Thus, we suspect that TGFBI is a potential target influenced by miR-21-5p.

### Binding Sites Between miR-21-5p and TGFBI and the Function of TGFBI in Pyroptosis

**Figure 5:**
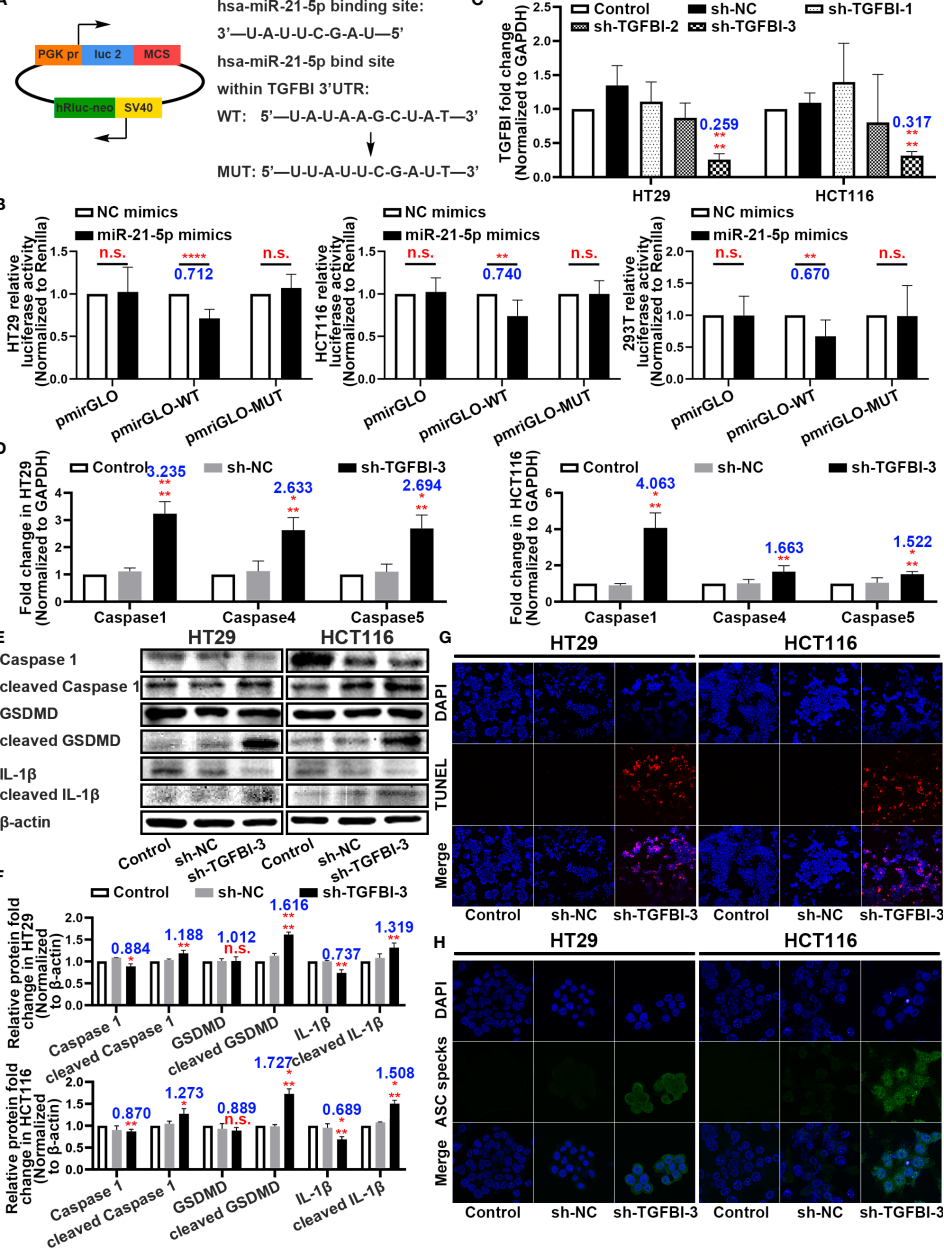
Binding sites between miR-21-5p and TGFBI and function research of TGFBI in pyroptosis. **(A)** Predicted binding sites for miR-21-5p on TGFBI and a diagram depicting the construction of the wild-type (WT) and mutant type (MUT) pmirGLO plasmids. **(B)** 293T, HT29, and HCT116 cells were co-transfected with miR-21-5p/NC mimics and pmirGLO/pmirGLO-WT/pmirGLO-MUT plasmids. Luciferase activity was detected 24 h after transfection using a dual-luciferase assay. **(C)** Validation of TGFBI sh-RNA knockdown efficiency in HT29 and HCT116 cells as determined by qRT-PCR. **(D)** Pyroptosis-associated mRNA levels in HT29 and HCT116 cells after sh-TGFBI-3 transfection as determined by qRT-PCR. **(E–F)** Expression of Pyroptosis-associated proteins in HT29 and HCT116 cells after sh-TGFBI-3 transfection as determined by western blot analysis. **(G)** TUNEL stain assay of cell death in sh-TGFBI-3 transferred HT29 and HCT116 cells. **(H)** Immunofluorescence staining of ASC specks expression in sh-TGFBI-3 transfected HT29 and HCT116 cells.The bars and error bars indicate the mean ± SD. n.s.p > 0.05, *p < 0.05, **p < 0.01, ***p < 0.005, ****p < 0.001.

With the rigorous bioinformatic prediction, we sub-cloned the wild-type (WT) or mutated-type (MUT) binding regions (predicted to have miR-21-5p binding sites; **Supplementary Table 3**) of *TGFBI* mRNA downstream of the firefly luciferase gene in the pmirGLO vector (**Figure 5A**). The evaluation of luciferase activity after co-transfection of cells with the miRNA mimics and luciferase plasmids revealed that overexpression of miR-21-5p significantly reduced the luciferase activity of pmirGLO-WT in the HT29, HCT116, and 293T cell lines (**Figure 5B**).

To further demonstrate the function of TGFBI, we constructed three short hairpin RNA plasmids to knockdown *TGFBI* (sequences are listed in **Supplementary Table 2**), and selected the knockdown efficiency of sh-TGFBI-3 as the best (**Figure 5C**). The qRT-PCR and western blot assays for HT29 and HCT116 cells with knockdown *TGFBI* revealed an overexpression of pyroptosis-associated mRNA (**Figure 5D**) and further cleavage of pyroptosis-related proteins (**Figures 5E, F**). Functionally, the TUNEL staining assay demonstrated that transfection of sh-TGFBI-3 exacerbated cell death (**Figure 5G**), while immunofluorescence of ASC specks demonstrated that cell death was primarily caused by pyroptosis (**Figure 5H**).

## Discussion

In this study, we compared miR-21-3p and miR-21-5p expression in CRC tissues and cell lines. The miR-21-5p levels in the patients’ tissues and HCT116 cells were significantly higher when compared to the other groups, however, the same effect was not observed in HT29 cells. This is consistent with the findings of a previous study (23). Surprisingly, we found that phenotypically CRC cell death was induced when the miR-21-3p inhibitor and miR-21-5p mimic/inhibitor were transfected into cells. In a previous CRC study, the apoptotic mechanism induced *via* inhibition of miR-21-3p was considered to be associated with multiple splicing through the Smad4/extra cellular signal-regulated protein kinase signalling pathway (24). There have also been various studies on miR-21-5p (25–27), however, currently the consensus is that repressing miR-21-5p suppresses proliferation and promotes apoptosis by modulating the PTEN/PI3K/AKT pathway (21, 22). Surprisingly, the miR-21-5p mimic also induced cell death and had a stronger effect than the miR-21-5p inhibitor. We, therefore, designed subsequent experiments based on these observations.

Various types of cell death have been discovered in cancers. The immunogenicity of cancer cells is an emerging determinant of anticancer immunotherapy (28). Recently, investigators have focused on the immunobiology of dying cancer cells and attempted to determine their relevance for the success of anticancer therapies (29–31). To verify whether miR-21-5p activates the immune responses associated with cell death, we investigated the levels of IL-1β and IL-18 and found that only miR-21-5p mimic upregulated inflammatory factors, suggesting that miR-21-5p-induced cell death occurs *via* a completely different mechanism from the apoptosis induced by the inhibitors. Interestingly, qRT-PCR revealed that caspase 1, caspase 4, and caspase 5 mRNA were upregulated, while western blot analysis showed that the caspase 1, GSDMD, and IL-1β proteins were cleaved; both of which indicate activation of the pyroptosis pathway.

Pyroptosis was previously referred to as caspase-1-induced PCD process (32). The induction factors of pyroptosis were regarded as immunopathological, including a range of microbial infections and non-infectious stimuli, such as host factors (33). The question then became how miR-21-5p induces pyroptosis in CRC cells. Accordingly, we screened the potential targets of miR-21-5p and used qRT-PCR to identify the genes with a target score ≥ 98. TGFBI was found to strongly follow the miRNA regulatory mechanism. Previously, TGFBI was considered to be a high-risk factor for increased incidence of spontaneous tumours in gastrointestinal tract cancers (34). In addition, mechanistic studies have shown that TGFBI is strongly associated with chemotherapy in NSCLC (35) and ovarian cancer (36). Similarly, the data from TCGA showed high expression of TGFBI in CRC patients, often resulting in low survival. In addition, western blot and immunofluorescence showed a high degree of consistency with the TGFBI qRT-PCR results, while dual-luciferase reporter assays in three cell lines suggested direct binding between miR-21-5p and mRNA of TGFBI.

As the nomenclature implies, TGFBI was first identified as a TGF-β-inducible gene (37), which has since been reported to regulate the premetastatic activity of TGF-β in some cancers (38, 39). In breast cancer research, distant metastases tend to develop when TGFBI and CD44 were expressed at high levels (40), which verifies the relationship between TGFBI and immune responses. Moreover, a previous report on CRC highlighted that TGFBI promotes metastasis by enhancing cell extravasation (41). In our study, with the establishment of the TGFBI-knockdown model, pyroptosis-associated mRNAs were upregulated and showed high levels of cleavage; whereas, the TUNEL assay further corroborated the presence of pyroptosis cell death.

However, pyroptosis cannot occur without inflammasome sensors. Inflammasome complexes become assembled upon activation of certain nucleotide-binding domain, leucine-rich repeat-containing proteins (NLR), AIM2-like receptors, or pyrin. Of these, NLRP1, NLRP3, NLRC4, NLRP6, and AIM2 influence the pathogenesis of cancer using various mechanisms of action (42). Specifically, NLRP3, which assembles the NLRP3-ASC-Caspase-1 inflammasome complex, activates pyroptosis of CRC cells (43). Meanwhile, the NAIP-NLRC4 inflammasome complex can also induce caspase-1-dependent cleavage of IL-1β, IL-18, and the pore-forming protein, gasdermin, D, leading to pyroptosis (44). However, recent studies demonstrated that the regulation and suppression of NLRC4 is not always consistent between cancer models or even within the same CRC model (45, 46). Still further, AIM2 has been reported as exhibiting tumour-suppressive functions in colon cancer (47) by suppressing the PI3K/AKT pathways to inhibit proliferation of colonic stem cells and promote cell death (48, 49). In our study, various inflammatory indicators, including IL-1β and IL-18 were upregulated and ACS specks were observed. These results provide evidence for the activation of the NLRP3-ASC-Caspase-1 inflammasome complex; however, the precise inflammasome sensor that becomes activated by miR-21-5p requires further exploration.

The first reported research on miR-21 in CRC was 13 years ago (50). Several clinical researchers have since performed miRNA microarray expression analysis between colon adenocarcinoma and normal tissues, and have reported high levels of miR-21 to be associated with poor survival and poor therapeutic outcome (51). Even the precursory clinical statistics of prognostic and predictive value of a miRNA signature in stage II colon cancer declared that miR-21-5p is strongly relevant to CRC (52). However, the precise role of miR-21-5p remains to be characterised. In other words, it is necessary to determine if miR-21-5p is simply a characteristic of tumours, or if it promotes CRC as the malignancy grade of CRC has been found to be positively correlated with the level of miR-21-5p.

In conclusion, our data demonstrated that overexpression of miR-21-5p could induce pyroptosis in CRC cells. Moreover, TGFBI may serve as an intermediate of this biological process. But the further mechanism of TGFBI in pyroptosis still unknown. Especially, the inflammasome sensor of the biological progress is the next focus point in our study. Furthermore, the mechanism certification on animal model and even on clinic is the next step on our scheme.

Combined with previous studies, we believe that miR-21-5p plays a distinct role in CRC. The sensitivity of CRC cells to the level of miR-21-5p is highly expressed *in vitro*. Thus, it might be suggested that miR-21-5p has great potential as a therapeutic target in CRC, however, the mechanism requires further elucidation.

## Data Availability Statement

The raw data supporting the conclusions of this article will be made available by the authors, without undue reservation.

## Ethics Statement

The studies involving human participants were reviewed and approved by Ethics Committee of Kunshan Hospital of Traditional Chinese Medicine. The patients/participants provided their written informed consent to participate in this study.

## Author Contributions

JX and JW conceived of the study and provided the project direction. RJ guided the experiments, analyzed the data, and wrote the manuscript. XC and SG completed the cell experiments. QW made contributions to the bioinformatics results. YL and HC read and revised the manuscript. All authors contributed to the article and approved the submitted version.

## Funding

This work was financially supported by the National Key Research and Development Program of China (2017YFC1703301), the National Natural Science Foundation of China (81873235 and 82003999), 1226 Major Project (BWS17J028), China Postdoctoral Science Foundation funded project (2020M681366), the Shanghai Post-doctoral Excellence Program (2019091), the Budget Internal Medicine Research Project of Shanghai University of Traditional Chinese Medicine (2020LK004), the Kunshan Social Development Science and Technology Project (KSZ1623).

## Conflict of Interest

The authors declare that the research was conducted in the absence of any commercial or financial relationships that could be construed as a potential conflict of interest.
