# Supplementary material for: MiR-21-5p Induces Pyroptosis in Colorectal Cancer via TGFBI
Source: Front Oncol. 2021 Feb 5;10:610545. doi: 10.3389/fonc.2020.610545 (PMC7892456; doi:10.3389/fonc.2020.610545)
Supplement: Supplementary file 1 [file Table_1.docx]

**Supplementary Table 1 The sequence of mimics and inhibitors.**

| **Name** | **Sequence（5’-3’）** | | **5’mark** | **Modification** |
| --- | --- | --- | --- | --- |
| hsa-miR-21-3p mimics | | CAACACCAGUCGAUGGGCUGU | N/A | N/A |
| hsa-miR-21-3p inhibitors | | ACAGCCCAUCGACUGGUGUUG | N/A | 2’-oMe |
| hsa-miR-21-5p mimics | | UAGCUUAUCAGACUGAUGUUGA | N/A | N/A |
| hsa-miR-21-5p inhibitors | | UCAACAUCAGUCUGAUAAGCUA | N/A | 2’-oMe |
| NC mimics | | UCACAACCUCCUAGAAAGAGUAGA | N/A | N/A |
| NC inhibitors | | UCUACUCUUUCUAGGAGGUUGUGA | N/A | 2’-oMe |

The sequence of hsa-miR-21-3p mimics and inhibitors, hsa-miR-21-5p mimics and inhibitors, NC mimics and inhibitors.
