# Supplementary material for: MiR-21-5p Induces Pyroptosis in Colorectal Cancer via TGFBI
Source: Front Oncol. 2021 Feb 5;10:610545. doi: 10.3389/fonc.2020.610545 (PMC7892456; doi:10.3389/fonc.2020.610545)
Supplement: Supplementary file 2 [file Table_2.docx]

**Supplementary Table 2 The sh-RNA sequence.**

| **Name** | **Oligo sequences** |
| --- | --- |
| Sh-NC-sense | 5'-gatctGTTCTCCGAACGTGTCACGTTTCAAGAGAACGTGA  CACGTTCGGAGAATTTTTTc-3 |
| Sh-NC-antisense | 5'- aattgAAAAAATTCTCCGAACGTGTCACGTTCTCTTGAAA  CGTGACACGTTCGGAGAACa-3’ |
| Sh-TGFBI-1-sense | 5'- GATCCGCATGACCCTCACCTCTATGTCTCGAGACATAG  AGGTGAGGGTCATGCTTTTTT-3’ |
| Sh-TGFBI-1-antisense | 5'- AATTAAAAAAGCATGACCCTCACCTCTATGTCTCGAGA  CATAGAGGTGAGGGTCATGCG-3’ |
| Sh-TGFBI-2-sense | 5'- GATCCGGGACATGCTCACTATCAACGCTCGAGCGTTGA  TAGTGAGCATGTCCCTTTTTT-3’ |
| Sh-TGFBI-2-antisense | 5'- AATTAAAAAAGGGACATGCTCACTATCAACGCTCGAG  CGTTGATAGTGAGCATGTCCCG-3’ |
| Sh-TGFBI-3-sense | 5'- GATCCGCTTCGGAACCACATAATTAACTCGAGTTAATT  ATGTGGTTCCGAAGCTTTTTT-3’ |
| Sh-TGFBI-3-antisense | 5'- AATTAAAAAAGCTTCGGAACCACATAATTAACTCGAGT  TAATTATGTGGTTCCGAAGCG-3’ |

The sense and antisense sequence of sh-TGFBI-1, sh-TGFBI-2, sh-TGFBI-3 and NC.
