# Supplementary material for: MiR-21-5p Induces Pyroptosis in Colorectal Cancer via TGFBI
Source: Front Oncol. 2021 Feb 5;10:610545. doi: 10.3389/fonc.2020.610545 (PMC7892456; doi:10.3389/fonc.2020.610545)
Supplement: Supplementary file 3 [file Table_3.docx]

**Supplementary Table 3 The dual-luciferase reporter plasmids.**

| **Name** | **Plasmids sequences** |
| --- | --- |
| PmirGLO-WT | GCTAGCCTTGAAGCACTACAGGAGGAATGCACCACGGCAGCTCTCCGCCAATTTCTCTCAGATTTCCACAGAGACTGTTTGAATGTTTTCAAAACCAAGTATCACACTTTAATGTACATGGGCCGCACCATAATGAGATGTGAGCCTTGTGCATGTGGGGGAGGAGGGAGAGAGATGTACTTTTTAAATCATGTTCCCCCTAAACATGGCTGTTAACCCACTGCATGCAGAAACTTGGATGTCACTGCCTGACATTCACTTCCAGAGAGGACCTATCCCAAATGTGGAATTGACTGCCTATGCCAAGTCCCTGGAAAAGGAGCTTCAGTATTGTGGGGCTCATAAAACATGAATCAAGCAATCCAGCCTCATGGGAAGTCCTGGCACAGTTTTTGTAAAGCCCTTGCACAGCTGGAGAAATGGCATCATT**ATAAGCTA**TGAGTTGAAATGTTCTGTCAAATGTGTCTCACATCTACACGTGGCTTGGAGGCTTTTATGGGGCCCTGTCCAGGTAGAAAAGAAATGGTATGTAGAGCTTAGATTTCCCTATTGTGACAGAGCCATGGTGTGTTTGTAATAATAAAACCAAAGAAACATATCTAGA |
| PmirGLO-MUT | GCTAGCCTTGAAGCACTACAGGAGGAATGCACCACGGCAGCTCTCCGCCAATTTCTCTCAGATTTCCACAGAGACTGTTTGAATGTTTTCAAAACCAAGTATCACACTTTAATGTACATGGGCCGCACCATAATGAGATGTGAGCCTTGTGCATGTGGGGGAGGAGGGAGAGAGATGTACTTTTTAAATCATGTTCCCCCTAAACATGGCTGTTAACCCACTGCATGCAGAAACTTGGATGTCACTGCCTGACATTCACTTCCAGAGAGGACCTATCCCAAATGTGGAATTGACTGCCTATGCCAAGTCCCTGGAAAAGGAGCTTCAGTATTGTGGGGCTCATAAAACATGAATCAAGCAATCCAGCCTCATGGGAAGTCCTGGCACAGTTTTTGTAAAGCCCTTGCACAGCTGGAGAAATGGCATCATT**TATTCGAT**TGAGTTGAAATGTTCTGTCAAATGTGTCTCACATCTACACGTGGCTTGGAGGCTTTTATGGGGCCCTGTCCAGGTAGAAAAGAAATGGTATGTAGAGCTTAGATTTCCCTATTGTGACAGAGCCATGGTGTGTTTGTAATAATAAAACCAAAGAAACATATCTAGA |

The dual-luciferase reporter plasmids sequence of PmirGLO-WT and PmirGLO-MUT.
