# Supplementary material for: MiR-21-5p Induces Pyroptosis in Colorectal Cancer via TGFBI
Source: Front Oncol. 2021 Feb 5;10:610545. doi: 10.3389/fonc.2020.610545 (PMC7892456; doi:10.3389/fonc.2020.610545)
Supplement: Supplementary file 4 [file Table_4.docx]

**Supplementary Table 4 Primers for qRT-PCR.**

| Gene | Primers for qRT-PCR |
| --- | --- |
| mir-21-3p-F | GCGCAACACCAGTCGATG |
| mir-21-3p-R | AGTGCAGGGTCCGAGGTATT |
| mir-21-3p-RT | GTCGTATCCAGTGCAGGGTCCGAGGTATTCGCACTGGATACGACACAGCC |
| mir-21-5p-F | GCGCGTAGCTTATCAGACTGA |
| mir-21-5p-R | AGTGCAGGGTCCGAGGTATT |
| mir-21-5p-RT | GTCGTATCCAGTGCAGGGTCCGAGGTATTCGCACTGGATACGACTCAACA |
| U6-F | AGAGAAGATTAGCATGGCCCCTG |
| U6-R | ATCCAGTGCAGGGTCCGAGG |
| U6-RT | GTCGTATCCAGTGCAGGGTCCGAGGTATTCGCACTGGATACGACAAAATA |
| Caspase1-F | CACACCGCCCAGAGCACAAG |
| Caspase1-R | TCCCACAAATGCCTTCCCGAATAC |
| Caspase4-F | GGAGGCTGGACCACCTGAGTC |
| Caspase4-R | AGGCGTGTGCGGTTGTTTCTC |
| Caspase5-F | TCCTAGAGGGAATCTGCGGAACTG |
| Caspase5-R | GCCTGGACAATGATGACCTTGGG |
| YOD1-F | GATGTCAACCGCTTCACCCTGAG |
| YOD1-R | CTGTCTCCTTGGCATGTTCCCTTG |
| FASLG-F | ACCGCCACCACTACCACCTC |
| FASLG-R | CCTACCAAGGCAACCAGAACCATG |
| PRDM11-F | GTACAAGCTCCGCATGCACC |
| PRDM11-R | CAGGTGGTAGGCGGTGTTGA |
| VCL-F | GCTCTGCTGATGGCTGAGATGTC |
| VCL-R | GGCGATGTCCTTGGCACACTG |
| ZNF367-F | GGAGGTGAGGACGAGGAGGAAG |
| ZNF367-R | ACGGATTCTGCTGGATGAATGCTC |
| SKP2-F | AGACTGGATGAGCTGAACCTCTCC |
| SKP2-R | GGTGATGGTCTCTGACACATGCG |
| TGFBI-F | CTGTGCCCGGCTGCTGAAAG |
| TGFBI-R | TCTGCTGGATGTTGTTGGTGATGG |
| GAPDH-F | CACCCACTCCTCCACCTTTG |
| GAPDH-R | CCACCACCCTGTTGCTGTAG |

The primers for qRT-PCR used in the article.
