# Supplementary material for: MiR-21-5p Induces Pyroptosis in Colorectal Cancer via TGFBI
Source: Front Oncol. 2021 Feb 5;10:610545. doi: 10.3389/fonc.2020.610545 (PMC7892456; doi:10.3389/fonc.2020.610545)
Supplement: Supplementary file 5 [file Table_5.docx]

**Supplementary Table 5 The bioinformatic prediction of targets (score≥90).**

| miRNA Name | Gene Symbol | Target Score |
| --- | --- | --- |
| hsa-miR-21-5p | YOD1 | 99 |
| hsa-miR-21-5p | FASLG | 99 |
| hsa-miR-21-5p | PRDM11 | 99 |
| hsa-miR-21-5p | VCL | 99 |
| hsa-miR-21-5p | ZNF367 | 99 |
| hsa-miR-21-5p | SKP2 | 98 |
| hsa-miR-21-5p | TGFBI | 98 |
| hsa-miR-21-5p | IL12A | 97 |
| hsa-miR-21-5p | RAB6D | 97 |
| hsa-miR-21-5p | ADGRG2 | 97 |
| hsa-miR-21-5p | RALGPS2 | 97 |
| hsa-miR-21-5p | PLAG1 | 97 |
| hsa-miR-21-5p | RBPJ | 97 |
| hsa-miR-21-5p | PELI1 | 97 |
| hsa-miR-21-5p | CREBRF | 97 |
| hsa-miR-21-5p | KRIT1 | 97 |
| hsa-miR-21-5p | SCML2 | 96 |
| hsa-miR-21-5p | RSAD2 | 96 |
| hsa-miR-21-5p | PBRM1 | 96 |
| hsa-miR-21-5p | GATAD2B | 96 |
| hsa-miR-21-5p | SPRY1 | 95 |
| hsa-miR-21-5p | PLEKHA1 | 95 |
| hsa-miR-21-5p | FGF18 | 95 |
| hsa-miR-21-5p | PPP1R3B | 95 |
| hsa-miR-21-5p | YAP1 | 94 |
| hsa-miR-21-5p | GPATCH2L | 94 |
| hsa-miR-21-5p | STAT3 | 94 |
| hsa-miR-21-5p | BCL7A | 94 |
| hsa-miR-21-5p | SKI | 94 |
| hsa-miR-21-5p | FAM13A | 94 |
| hsa-miR-21-5p | MALT1 | 94 |
| hsa-miR-21-5p | ZBTB41 | 93 |
| hsa-miR-21-5p | KDM7A | 93 |
| hsa-miR-21-5p | MBNL3 | 93 |
| hsa-miR-21-5p | CCL1 | 93 |
| hsa-miR-21-5p | NKIRAS1 | 93 |
| hsa-miR-21-5p | TIAM1 | 93 |
| hsa-miR-21-5p | OSR1 | 93 |
| hsa-miR-21-5p | KLF3 | 93 |
| hsa-miR-21-5p | PAN3 | 93 |
| hsa-miR-21-5p | PDCD4 | 92 |
| hsa-miR-21-5p | AKAP12 | 92 |
| hsa-miR-21-5p | GID4 | 92 |
| hsa-miR-21-5p | HSD17B4 | 92 |
| hsa-miR-21-5p | PDZD2 | 92 |
| hsa-miR-21-5p | CPEB3 | 92 |
| hsa-miR-21-5p | CASKIN1 | 92 |
| hsa-miR-21-5p | MAP3K1 | 92 |
| hsa-miR-21-5p | UBE2D3 | 92 |
| hsa-miR-21-5p | NTF3 | 91 |
| hsa-miR-21-5p | TIMP3 | 91 |
| hsa-miR-21-5p | RECK | 91 |
| hsa-miR-21-5p | CCL20 | 91 |
| hsa-miR-21-5p | JAG1 | 91 |
| hsa-miR-21-5p | ANGPTL5 | 91 |
| hsa-miR-21-5p | PPP1R3A | 91 |
| hsa-miR-21-5p | BCL11B | 91 |
| hsa-miR-21-5p | BTG2 | 90 |
| hsa-miR-21-5p | LRRC57 | 90 |
| hsa-miR-21-5p | NFIA | 90 |
| hsa-miR-21-5p | MPRIP | 90 |
| hsa-miR-21-5p | SLC30A10 | 90 |
| hsa-miR-21-5p | SYT15 | 90 |
| hsa-miR-21-5p | MEI4 | 90 |
| hsa-miR-21-5p | GLCCI1 | 90 |
| hsa-miR-21-5p | KLHL15 | 90 |
| hsa-miR-21-5p | CFAP300 | 90 |
| hsa-miR-21-5p | FAM3C | 90 |
| hsa-miR-21-5p | EPM2A | 90 |
| hsa-miR-21-5p | SPRY2 | 90 |

The bioinformatic prediction of downstream targets of hsa-miR-21-5p (score≥90).
