# Supplementary material for: MiR-21-5p Induces Pyroptosis in Colorectal Cancer via TGFBI
Source: Front Oncol. 2021 Feb 5;10:610545. doi: 10.3389/fonc.2020.610545 (PMC7892456; doi:10.3389/fonc.2020.610545)
Supplement: Supplementary file 6 [file Table_6.docx]

**Supplementary Table 6 Basic information of the patients.**

| **Patient number** | **Male/Femal** | **Age** | **Clinical stages** |
| --- | --- | --- | --- |
| 201719513 | male | 53 | T4bN0M0, IIc |
| 201803924 | female | 43 | T4bN0M1a, IVa |
| 201916835 | male | 64 | T4bN1M0, IIIc |
| 201919498 | female | 49 | T4bN2M0, IIIc |
| 202003884 | female | 83 | T4bN0M0, IIc |

Basic information of the patients from clinic.
